# Dissecting the genetic relationship between severe mental disorders and autoimmune diseases

**DOI:** 10.1016/j.bbi.2025.106249

**Published:** 2025-12-28

**Authors:** Erik D. Wiström, Julian Fuhrer, Alexey Shadrin, Vera Fominykh, Kevin S. O’Connell, Piotr Jaholkowski, Nils Eiel Steen, Sara E. Stinson, Pravesh Parekh, Oleksandr Frei, Linn Rødevand, Nadine Parker, Jan Haavik, Srdjan Djurovic, Anders M. Dale, Ole A. Andreassen, Olav B. Smeland

**Affiliations:** a Centre for Precision Psychiatry, Institute of Clinical Medicine, University of Oslo, Oslo, Norway; b Division of Mental Health and Addiction, Oslo University Hospital, Oslo, Norway; c Division of Mental Health and Substance Abuse, Diakonhjemmet Hospital, Oslo, Norway; d Department of Biomedicine, Faculty of Medicine, University of Bergen, Bergen, Norway; e Division of Psychiatry, Haukeland University Hospital, Bergen, Norway; f Department of Medical Genetics, Oslo University Hospital, Oslo, Norway; g Department of Clinical Science, University of Bergen, Bergen, Norway; h Center for Multimodal Imaging and Genetics, J. Craig Venter Institute, La Jolla, CA, USA; i University of California San Diego, La Jolla, CA, USA; j Oslo University Hospital, Oslo, Norway

## Abstract

Severe mental disorders have been linked to immune system dysfunction. While a genetic association between mental disorders and autoimmune diseases has been suggested, their genetic relationship remains incompletely understood. Utilizing a complementary set of statistical analyses, we conducted a comprehensive investigation of the genetic architecture between severe mental disorders (major depression (MD), bipolar disorder (BD), and schizophrenia (SCZ)) and seven autoimmune diseases (autoimmune thyroiditis, celiac disease, inflammatory bowel disease (IBD), multiple sclerosis, psoriasis, rheumatoid arthritis, and type 1 diabetes), involving a total of 667,518 cases from 10 genome-wide association studies. While MD was positively genetically correlated with five autoimmune diseases, BD and SCZ were only positively correlated with IBD, suggesting differences in the genetic signal shared with autoimmunity across these mental disorders. A considerable fraction of genetic variants influencing autoimmune diseases (range 17.1–88.4 %) was estimated to overlap with mental disorders; however, this constitutes only a minor part of genetic variants influencing the more polygenic mental disorders. Finally, we identified 172 genetic loci jointly affecting mental disorders and autoimmune diseases, implicating both lipid metabolism and TNF signaling. In conclusion, MD, BD, and SCZ have a small but distinct genetic overlap with autoimmune diseases, which may inform new possible immune targets for treatment in mental illness.

## 1. Introduction

There is a growing interest in the relationship between severe mental disorders and the immune system, with evidence suggesting that immune processes may contribute to psychiatric pathophysiology (Bennett and Molofsky, 2019). Major depression (MD), bipolar disorder (BD), and schizophrenia (SCZ) have all been linked to elevated markers of inflammation in serum (Ermakov et al., 2022; Poletti et al., 2024), as well as in the cerebrospinal fluid (Orlovska-Waast et al., 2019). Further, there is a proposed link between infection and these mental disorders (Yolken and Torrey, 2008; Wang et al., 2014; Oliveira et al., 2017), mirroring the well-established link between infections and the onset of autoimmune disease (Getts et al., 2013).

A key area of current research is the potential involvement of autoimmune mechanisms in psychiatric conditions. Psychiatric symptoms such as depression, anxiety, and paranoia are frequently observed in autoimmune diseases, including multiple sclerosis (MS), autoimmune thyroiditis (AITD), and inflammatory bowel disease (IBD) (Sarısoy et al., 2013; Leyhe and Müssig, 2014; Kurina et al., 2001). Moreover, some studies show that individuals with autoimmune diseases are at increased risk of developing MD, BD, and SCZ compared to the general population (Euesden et al., 2017; Chen et al., 2021; Jeppesen and Benros, 2019). For instance, a 2019 meta-analysis, combining eight separate studies, found that individuals with non-neurological autoimmune diseases had higher odds of subsequently being diagnosed with a psychotic disorder (odds ratio = 1.44, confidence interval, 1.04–1.95) (Cullen et al., 2019). Notably, this relationship appears to be bidirectional, whereby mental disorders similarly predispose individuals to the later onset of autoimmune disease (Euesden et al., 2017; Chen et al., 2021; Jeppesen and Benros, 2019; Cullen et al., 2019; Marrie et al., 2019).

Given that both groups of conditions are heritable, an important unresolved question is whether shared genetic mechanisms contribute to these observed associations (Pisetsky, 2023; Baselmans et al., 2021). Several studies have reported positive genetic correlations between MD, BD, and SCZ and a variety of autoimmune diseases, such as IBD, psoriasis (PS), celiac disease (CeD), and MS (Tylee et al., 2018; Smeland et al., 2025; Levey et al., 2021). One study found that polygenic risk scores for SCZ predicted disease status in Crohn’s disease, type 1 diabetes (T1D), and rheumatoid arthritis (RA) (Stringer et al., 2014). However, not all studies support a genetic association. A recent genome-wide association study (GWAS) on BD did not find any significant genetic correlations with autoimmune diseases (Mullins et al., 2021), while another study found a small, but significant negative genetic correlation between SCZ and RA (Lee et al., 2015). Furthermore, while GWASs on MD, BD, and SCZ have identified genomic loci that are associated with both mental disorders and immune mechanisms (O’Connell et al., 2025; Trubetskoy et al., 2022; Adams et al., 2025), the specific genetic mechanisms that might underlie immunological abnormalities in severe mental disorders remain largely unknown. Clarifying these mechanisms might inform underlying psychopathology and lead to novel strategies for treatment and precision medicine development in psychiatry.

Genetic pleiotropy refers to genetic variants that affect two or more traits simultaneously (Solovieff et al., 2013). Genetic pleiotropy has mainly been assessed at the local level, focusing on individual genes or loci identified at the genome-wide significance threshold, or at the global level, using tools like linkage disequilibrium score regression (LDSC) that estimate the correlation in all SNP effects across the genome between two traits (Bulik-Sullivan et al., 2015). However, advances in statistical analysis of large-scale GWAS data have introduced new ways to study pleiotropy, demonstrating that the genetic relationships between complex human traits and disorders are more complex than identified using standard tools (Smeland et al., 2025; Hindley et al., 2022; Werme et al., 2022). For example, the bivariate causal mixture model (MiXeR) (Frei et al., 2019); Local Analysis of [co]Variant Association (LAVA) (Werme et al., 2022), and conjunctional false discovery rate (conjFDR) (Smeland et al., 2020) provide complementary measures of genetic overlap that may elucidate important aspects of the shared genetic architecture of polygenic phenotypes. Specifically, bivariate MiXeR quantifies the number of unique and overlapping genetic variants influencing two traits (Frei et al., 2019), LAVA identifies local regions in the genome with significantly correlated genetic signals (Werme et al., 2022), while conjFDR analysis increases power to discover individual trait-associated SNPs by leveraging overlapping genetic associations between two GWASs (Smeland et al., 2020; Andreassen et al., 2013).

**Fig. 1.**
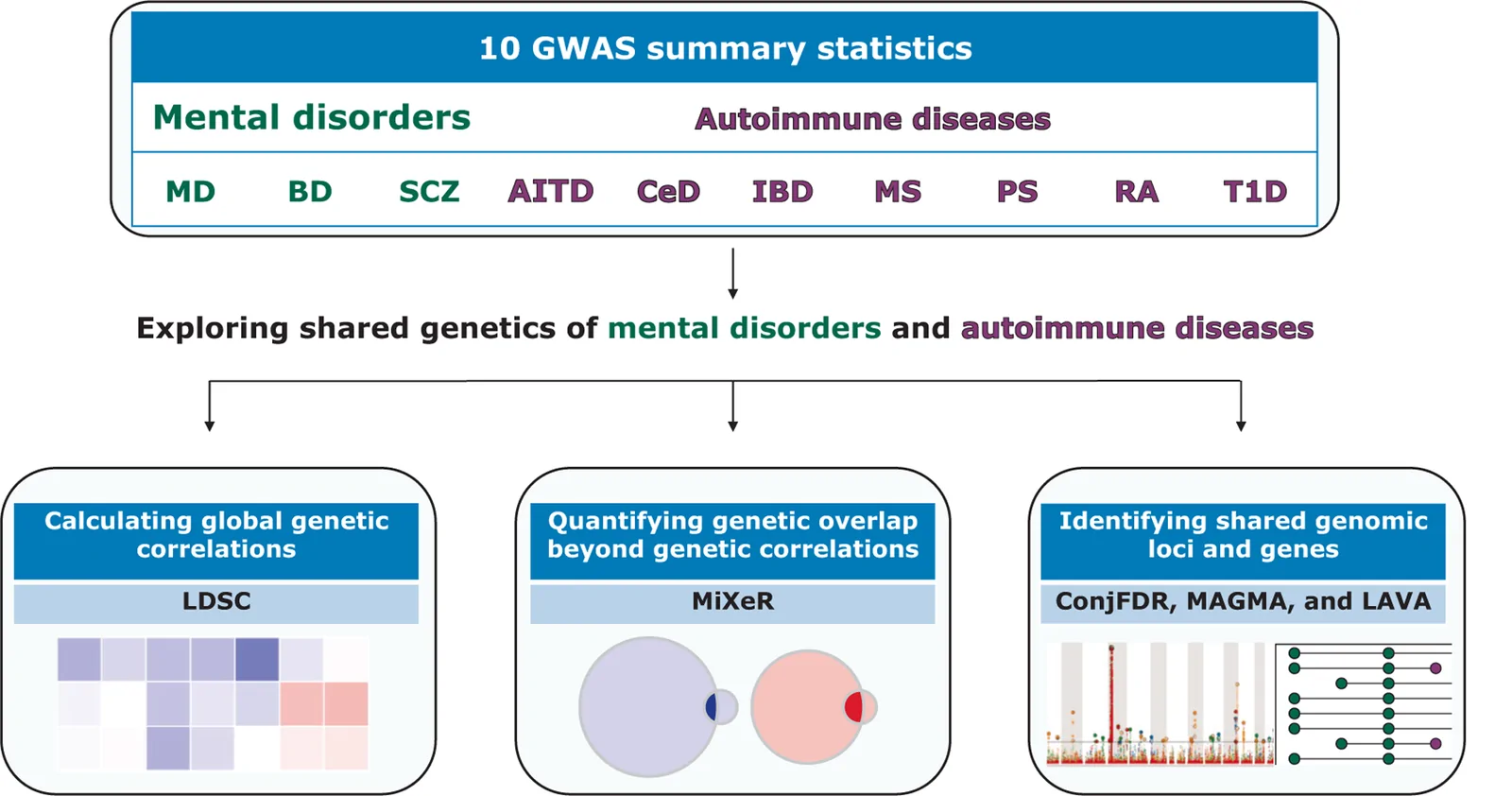
Study design overview.

In this genetic study, we aimed to comprehensively dissect the genetic relationship between the severe mental disorders SCZ, BD, and MD and common autoimmune diseases, to pinpoint potentially shared molecular genetic mechanisms and to investigate the overlapping and distinct genetic architectures across these disorders. We analyzed the largest collection of GWAS summary statistics on these phenotypes to date, and applied the statistical methods LDSC (Bulik-Sullivan et al., 2015); MiXeR (Frei et al., 2019); conjFDR (Smeland et al., 2020); Multivariate Analysis of Genomic Annotation (MAGMA) (de Leeuw et al., 2015), and LAVA to investigate complementary aspects of genetic pleiotropy (Werme et al., 2022). For an overview of study design, see Fig. 1.

Overview of the genome-wide association study (GWAS) summary statistics and analyses performed in the study. Abbreviations of mental disorders: major depression (MD), bipolar disorder (BD), and schizophrenia (SCZ). Abbreviations of autoimmune diseases: autoimmune thyroiditis (AITD), celiac disease (CeD), inflammatory bowel disease (IBD), multiple sclerosis (MS), psoriasis (PS), rheumatoid arthritis (RA), and type 1 diabetes (T1D). Abbreviations statistical methods: linkage disequilibrium score regression (LDSC), bivariate causal mixture model (MiXeR), conjunctional false discovery rate (conjFDR), Multivariate Analysis of Genomic Annotation (MAGMA), and Local Analysis of [co] Variant Association (LAVA).

## 2. Material and methods

### 2.1. Participant samples and phenotypes

We collected publicly available GWAS summary statistics on the severe mental disorders MD, BD, and SCZ, totaling 524,978 cases (O’Connell et al., 2025; Trubetskoy et al., 2022; Adams et al., 2025). For autoimmune diseases, we focused on a broad set of conditions that affect different organ systems, with different involvement of the autoimmune-autoinflammatory axis (Fominykh et al., xxxx; Breunig et al., 2025), including AITD, CeD, IBD, MS, PS, RA, and T1D. We collected GWAS summary statistics from repositories and published studies. For CeD and PS, we performed GWAS meta-analyses utilizing data from UK Biobank, FinnGen, the Norwegian Mother, Father and Child Cohort Study (MoBa), and the Hordaland Health Study (HUSK) (Magnus et al., 2016; Refsum et al., 2006; Paltiel et al., 2014; Kurki et al., 2023), to increase power for subsequent analyses, see Supplementary Note. Cases for autoimmune diseases collectively comprised 142,540 individuals (Saevarsdottir et al., 2020; de Lange et al., 2017; International Multiple Sclerosis Genetics Consortium, 2019; Ishigaki et al., 2022; Chiou et al., 2021). For an overview of cases and controls for each phenotype, see Table 1.

To avoid sample overlap in our conjFDR analyses, we removed cohorts from GWAS summary statistics on the mental disorder that were also included in the autoimmune GWASs. For more details on phenotype definition, diagnostic criteria, and country of origin, see Supplementary Note. Informed consent was gathered from each participant in the original studies. All participants had European ancestry, which ensured comparable LD patterns for follow-up analyses.

### 2.2. Statistical analysis

#### 2.2.1. Study design

After harmonizing and pre-processing of the GWAS summary statistics, we conducted a comprehensive analysis of the shared genetic signal between mental and autoimmune disorders at both the global and local level. To this end, we used a complementary set of statistical methods with different techniques and modeling assumptions. To quantify genome-wide genetic overlap, we applied LDSC to estimate global genetic correlations and MiXeR to quantify the number of shared genetic variants regardless of the global genetic correlation (Bulik-Sullivan et al., 2015; Frei et al., 2019). To investigate local overlap, we applied LAVA to quantify the local genetic correlation in pre-specified regions in the genome (Werme et al., 2022); conjFDR to increase statistical power to detect shared genomic loci (Smeland et al., 2020), and finally MAGMA to identify genome-wide significant shared genes (de Leeuw et al., 2015). We also biologically interrogated the shared genomic loci and genes. Note that the extended major histocompatibility complex (MHC) region has previously been strongly associated with both psychiatric and autoimmune disorders (Bennett and Molofsky, 2019; Pisetsky, 2023), but given its intricate LD structure, its inclusion in statistical analyses may potentially bias estimates of genetic overlap (Frei et al., 2019; Smeland et al., 2020; de Leeuw et al., 2015). To avoid this, we applied the default recommended settings for each method with regards to the MHC region. Specifically, the region was included for LDSC and LAVA analyses but excluded for the MAGMA and MiXeR analyses. For conjFDR analyses, it was excluded from the fitting of the FDR model but retained in the discovery phase following standard practice (Bulik-Sullivan et al., 2015; Werme et al., 2022; Smeland et al., 2020; de Leeuw et al., 2015; van der Meer et al., 2025).

### 2.3. Assessment of global genetic overlap

#### 2.3.1. Calculating genome-wide genetic correlations

We employed bivariate LDSC based on HapMap 3 SNPs to estimate genetic correlations between severe mental disorders and autoimmune diseases (Bulik-Sullivan et al., 2015). To account for multiple testing, we applied the false discovery rate (FDR) procedure with a significance threshold of 0.05. SNPs within the MHC region were included, following the default procedure in these analyses. Given that MD exhibited positive genetic correlations with a greater number of autoimmune diseases than BD and SCZ, we conducted a sensitivity analysis assessing the potential impact of comorbid MD on these results using UK Biobank data. Specifically, we repeated the LDSC analyses between MD and the autoimmune diseases AITD, CeD, IBD, MS, and PS before and after excluding MD cases from the autoimmune disease UK Biobank datasets, corrected for multiple comparisons using FDR.

#### 2.3.2. Quantifying polygenic overlap beyond genetic correlations

We applied the Gaussian mixture models incorporated in MiXeR version 1.3 (Frei et al., 2019; van der Meer et al., 2025; Holland et al., 2020). First, we conducted a univariate MiXeR analysis calculating the polygenicity (i.e., the number of genetic variants influencing a trait) and the discoverability (i.e., the average magnitude of effect sizes of the trait-influencing variants) for each mental disorder and autoimmune disease. The model estimates the number of variants that collectively account for 90 % of the SNP heritability (*h*^2^_*SNP*_), excluding those with negligibly small effect sizes to ensure the robustness of the estimate. We then performed bivariate MiXeR to estimate the amount of overlap between trait-influencing variants affecting the phenotypes. We conducted 20 iterations of MiXeR analysis with 2 million random SNPs followed by random pruning at an r^2^ threshold of 0.8. The extended MHC region (genome build 19, location 26,000,000–34,000,000) was excluded due to its complex LD structure, following the default procedure.

We assessed model fit for univariate MiXeR by comparing Akaike information criterion (AIC) values between the MiXeR estimates and a reference model where all variants are assumed to be causal (infinitesimal model). A positive difference in AIC between MiXeR estimates and reference model indicates that the best-fitting MiXeR estimates are more accurate than the reference model, thus supporting the validity of the analysis. For bivariate MiXeR, the model fit was similarly evaluated by comparing the best-fitting MiXeR model against two reference models representing minimum and maximum possible overlap respectively. We present conditional quantile–quantile (Q-Q) plots and log-likelihood plots to visualize the stability of the fitness procedure.

### 2.4. Assessment of local genetic overlap

#### 2.4.1. Investigating local genetic correlations

We applied LAVA v. 1.3.8 (Werme et al., 2022) to identify specific genomic regions exhibiting local genetic correlations between mental disorders and autoimmune diseases. Following the standard LAVA procedure, the genome was divided into 2,495 regions of approximately 1 Mb in size, with the LD reference panel constructed based on individuals of European ancestry from the 1000 Genomes phase 3 data. LAVA accounts for potential sample overlap using LDSC. After estimating the local *h*^2^_*SNP*_ for each phenotype, we conducted pairwise local genetic correlation analysis for all regions with local *h*^2^_*SNP*_ significantly different from zero. We utilized FDR correction to account for multiple testing. Furthermore, we compared the number of significant local genetic correlations within and outside of the extended MHC, defined according to the method's recommendations (genome build 19, location 26,000,000–34,000,000).

#### 2.4.2. Mapping shared genomic loci and genes

To increase statistical power to discover genetic loci that jointly affect severe mental disorders and autoimmune diseases, we used the conjFDR method (Smeland et al., 2020; Andreassen et al., 2013). To control for spurious enrichment, we calculated average empirical distribution functions by randomly selecting one SNP in each LD block (r^2^ > 0.1) over 500 iterations. As is standard for conjFDR analysis, the extended MHC (genome build 19, location 25,119,106–33,854,733) and the chromosomal region 8p23.1 (location 7,200,000–12,500,000) were excluded before fitting the FDR model, to avoid potential inflation of the FDR estimates (Smeland et al., 2020). However, note that the extended MHC and 8p23,1 regions are included in the subsequent discovery phase to allow detection of potentially shared associations with these regions (Smeland et al., 2025; Smeland et al., 2020; Smeland et al., 2021).

We utilized the FUMA protocol to define genomic loci (Watanabe et al., 2017). For linking lead SNPs to the most likely causal genes, we used the open web platform Open Targets Genetics (https://genetics.opentargets.org/) (Ghoussaini et al., 2021). Functional information on these genes was retrieved from GeneCards (Stelzer et al., 2016). We performed gene expression and gene-set analysis applying FUMA and Genotype-Tissue Expression (GTEx) dataset v8 on the genes identified by Open Targets, excluding lead SNPs located in the extended MHC region. We performed three sets of analyses with genes associated with MD, BD, and SCZ respectively. For more details, see Supplementary Note.

#### 2.4.3. Identifying genome-wide significant shared genes

Complementary to the conjFDR procedure, we also utilized MAGMA version 1.08 (reference dataset 1,000 Genomes European panel) (de Leeuw et al., 2015) to identify genome-wide significant genes shared with psychiatric disorders and autoimmune diseases. First, we identified likely causal genes for each GWAS and subsequently evaluated overlap of identified genes across psychiatric and autoimmune phenotypes. Functional annotations of selected genes were retrieved from GeneCards (Stelzer et al., 2016). MAGMA calculates an association *P* value for each gene based on all SNPs mapped to the gene, accounting for gene size, number of SNPs within each gene, and LD between markers. We excluded the extended MHC from the analysis (genome build 19, location 26,000,000–34,000,000) as this is the default recommendation for the method. We set the significance threshold to 0.05 after Bonferroni correction for multiple testing.

## 3. Results

### 3.1. Global genetic overlap

#### 3.1.1. Global genetic correlations

**Fig. 2.**
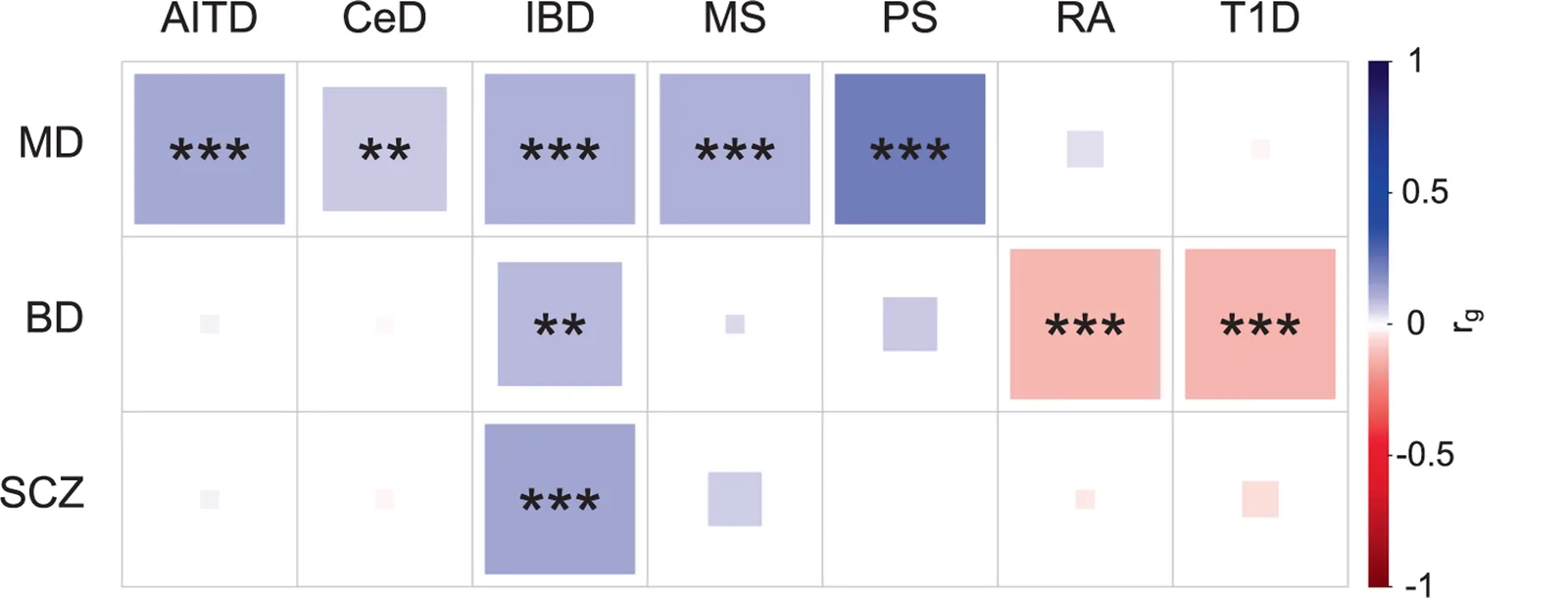
Global genetic correlations from LDSC analyses.

Our LDSC analyses revealed different patterns of global genetic correlations among MD, BD, and SCZ with autoimmune diseases (Fig. 2, Supplementary Table 1). While MD was significantly positively correlated with five autoimmune diseases (AITD, CeD, IBD, MS, and PS; r_g_ range 0.06–0.22), BD and SCZ were only positively correlated with IBD (0.10 and 0.13, respectively). Additionally, BD had significant negative correlations with RA and T1D (− 0.12 and − 0.13, respectively); no other genetic correlations were statistically significant after FDR correction. The genetic correlations within the two diagnostic categories are presented in Supplementary Fig. 1 and Supplementary Table 2.

Global genetic correlations observed between the psychiatric disorders major depression (MD), bipolar disorder (BD), and schizophrenia (SCZ), and the autoimmune diseases autoimmune thyroiditis (AITD), celiac disease (CeD), inflammatory bowel disease (IBD), multiple sclerosis (MS), psoriasis (PS), rheumatoid arthritis (RA), and type 1 diabetes (T1D). Colors represent r_g_ values, and the size of the colored square reflects the magnitude of the *P* value. Statistically significant correlations after false discovery rate (FDR) correction are denoted by asterisks: * FDR *P* < 0.05; ** FDR *P* < 0.01, *** FDR *P* < 0.001.

To investigate whether the significant positive correlations between MD and autoimmune diseases could be driven by the presence of cases with comorbid MD among patients with autoimmune diseases, we repeated LDSC correlation analyses using UK Biobank data to generate autoimmune disease GWAS where we could exclude psychiatric diagnoses. Subsequently, we compared the LDSC results when including and excluding cases with comorbid MD using this dataset. While the LDSC analyses for AITD and MS became invalid due to non-significant *h*^2^_*SNP*_ estimates, the other correlation estimates for CeD, IBD, and PS remained consistently positively correlated, though slightly attenuated (Pearson calculation = 0.9994, *P* value 0.02; Supplementary Table 3).

#### 3.1.2. Genome-wide genetic overlap beyond global genetic correlations

**Fig. 3.**
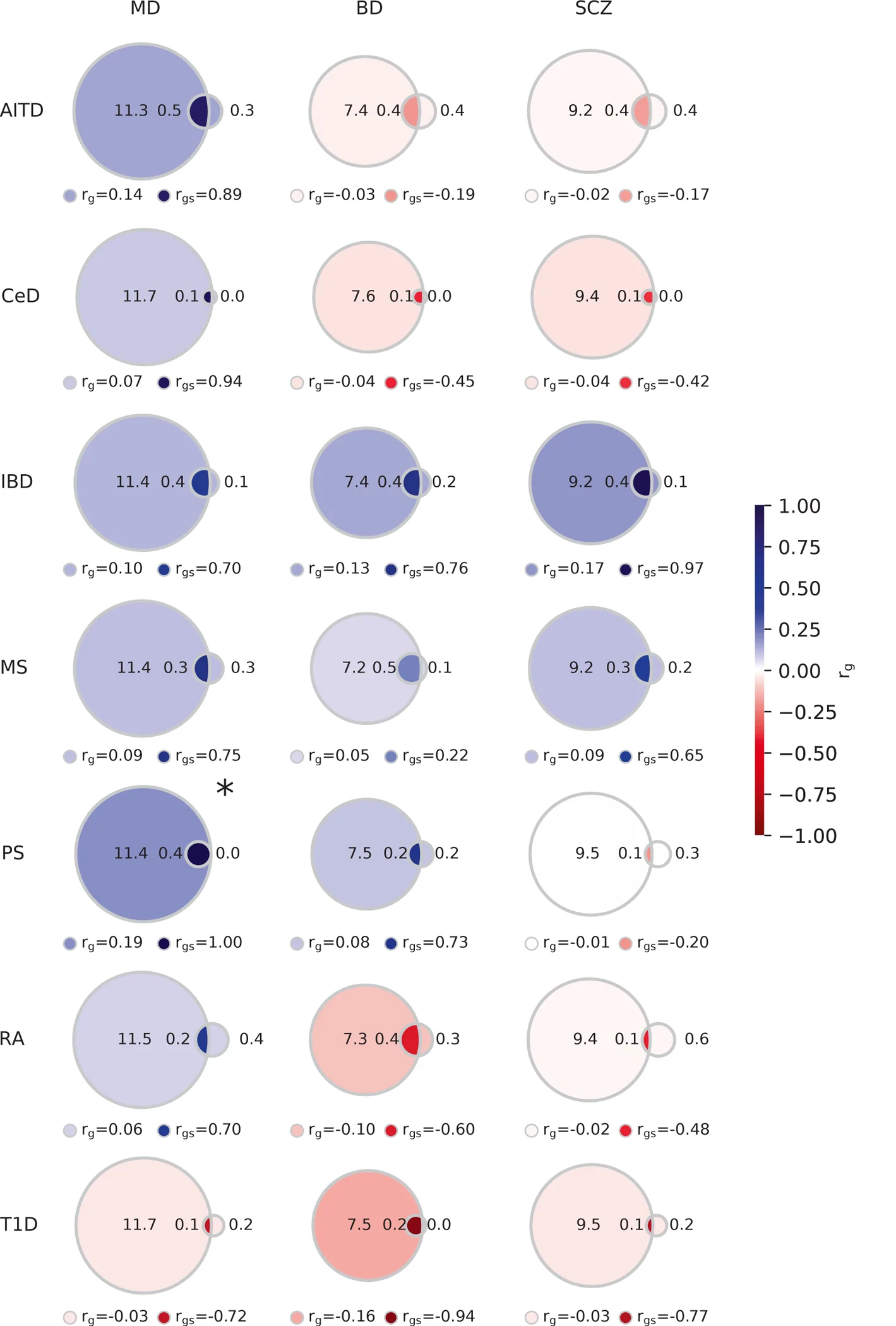
Genetic overlap estimated by MiXeR.

All univariate MiXeR analyses exhibited positive AIC values, indicating good model fit (Supplementary Table 4). We observed considerable differences in the estimated polygenicity across the two groups of conditions. The estimated number of trait-influencing variants ranged from 8.1 K to 11.8 K for severe mental disorders and from 0.13 K to 0.73 K for autoimmune diseases. Bivariate MiXeR revealed considerable overlap of genetic variants influencing psychiatric and autoimmune phenotypes, also in pairings where LDSC analysis showed no genetic correlation. We observed differences in the proportion of autoimmune disease variants overlapping with mental disorders. In particular, MS, PS, RA, and T1D displayed a larger degree of overlap with BD than with MD or SCZ. The proportion of trait-influencing variants shared with BD ranged from 50.6 % of trait-influencing AITD variants to 88.4 % of trait-influencing T1D variants (Fig. 3). For MD versus autoimmune diseases, T1D shared the lowest proportion of its variants (34.6 %), while IBD shared the highest proportion of its variants (73.6 %). For SCZ versus autoimmune diseases, RA shared the lowest proportion of its variants (17.1 %) and CeD shared the highest proportion (87.3 %). Mirroring the LDSC results, the effect directions of the shared variants were positive between MD and all autoimmune diseases except T1D, whereas BD and SCZ exhibited more mixed effect directions. For bivariate MiXeR, comparisons of the best estimates to the minimum AIC yielded positive values for all analyses except for MD and PS, for which the analysis was unable to precisely estimate the number of shared variants (Supplementary Table 5, Supplementary Fig. 2). For MiXeR estimates of required effective sample sizes to explain a specific percentage of *h*^2^_*SNP*_ by genome-wide significant variants, see Supplementary Fig. 3.

Genetic overlap quantified by bivariate MiXeR analyses of the psychiatric disorders major depression (MD), bipolar disorder (BD), and schizophrenia (SCZ), and the autoimmune diseases autoimmune thyroiditis (AITD), celiac disease (CeD), inflammatory bowel disease (IBD), multiple sclerosis (MS), psoriasis (PS), rheumatoid arthritis (RA), and type 1 diabetes (T1D). Each bivariate MiXeR analysis is illustrated with the psychiatric disorder as the circle on the left, and the autoimmune disease as the circle on the right. The area where the circles overlap represents the number of shared genetic variants between the traits. Numbers within and adjacent to the circles indicate an estimate of trait-influencing variants in the thousands. Below are estimates of genetic correlations between the phenotypes, as well as genetic correlations of the shared variants. *In the MD and PS analysis, comparisons of the best estimate to the minimum Akaike information criterion (AIC) yielded a negative value, meaning that the analysis was unable to precisely estimate the number of shared variants.

### 3.2. Overlap in genomic loci and genes

#### 3.2.1. Local genetic correlations

In line with the MiXeR findings of wide-spread genetic overlap, we identified multiple genomic regions exhibiting significant local genetic correlations between severe mental disorders and autoimmune diseases using LAVA, irrespective of the global genetic correlations between traits (Supplementary Table 6). For all mental disorders, we identified more regions with local correlations outside the MHC (105 for MD, 93 for BD, 144 for SCZ) than inside the MHC (11 for MD, 14 for BD, 19 for SCZ) (Supplementary Fig. 4), despite the well-established role of this locus in both psychiatric and autoimmune conditions (Pisetsky, 2023; O’Connell et al., 2025; Trubetskoy et al., 2022; Adams et al., 2025). We also identified the most pleiotropic genomic hotspots, with 18 regions displaying significant local correlations involving at least three conditions (Supplementary Table 7). Among these, 12 were located within the extended MHC.

#### 3.2.2. Discovery of shared genomic loci

**Fig. 4.**
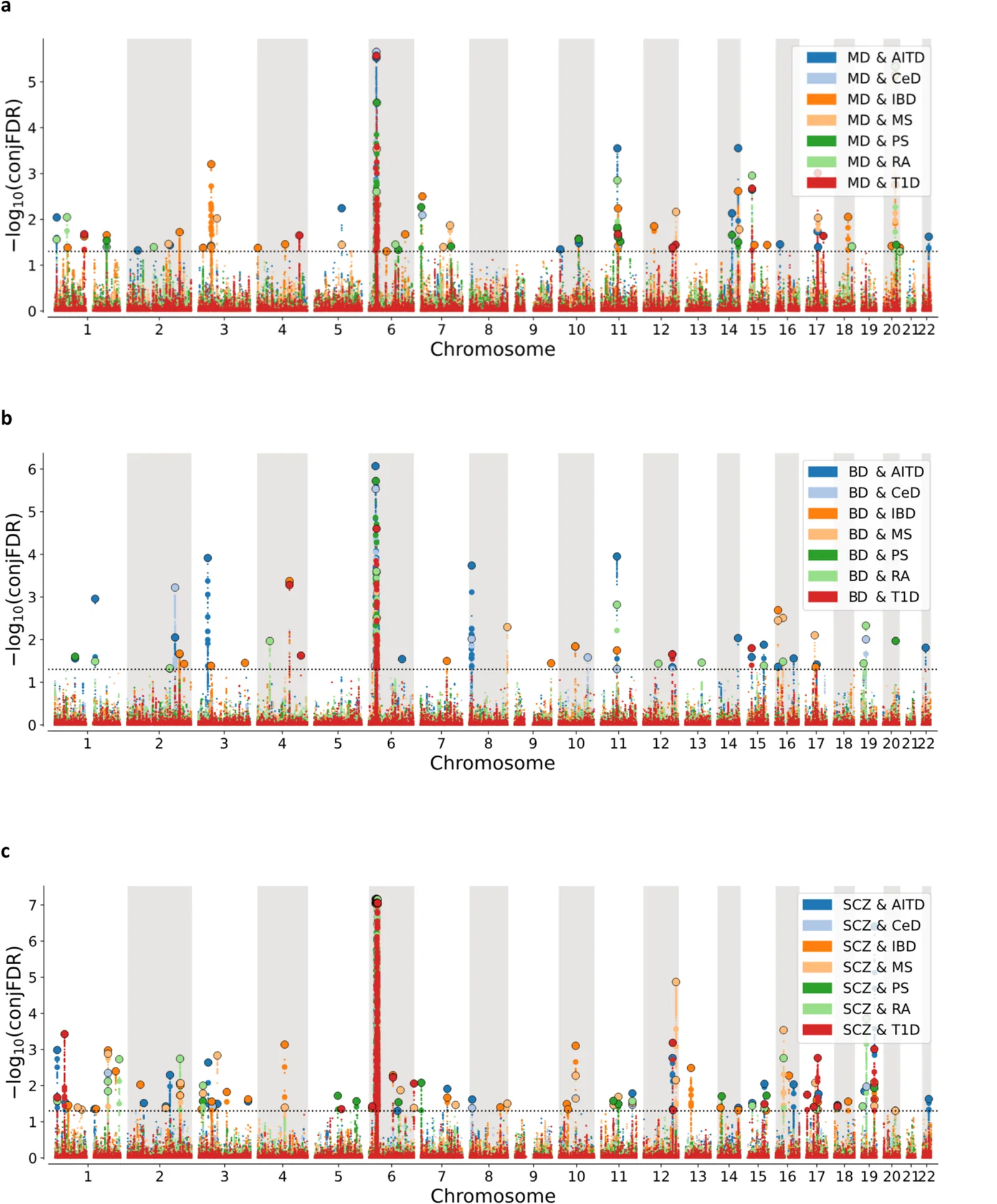
Miami plots of shared genome-wide significant loci identified by conjFDR.

The conditional Q-Q plots displayed an enrichment of SNP associations with mental disorders as a function of increasing levels of SNP associations with autoimmune diseases, and *vice versa* (Supplementary Fig. 5), corroborating the findings of widespread genetic pleiotropy between mental disorders and autoimmune diseases. We leveraged this cross-trait enrichment using conjFDR and identified 47 unique genetic loci associated with MD and at least one autoimmune disease, 14 of which were not found in the original GWAS, 38 unique loci associated with BD, 22 of which were not found in the original GWAS, and 87 unique loci associated with SCZ, 40 of which were not found in the original GWAS (Fig. 4 and Supplementary Tables 8–28). Each conjFDR analysis between a mental disorder and an autoimmune disease implicated the extended MHC region. Outside of the MHC, 27 loci overlapped with two mental disorders, while only three loci were associated with all mental disorders.

To further explore the potential biological mechanisms involved in the observed genetic pleiotropy between psychiatric and autoimmune conditions, we identified the most likely causal gene for the identified loci using Open Targets, see Supplementary Table 29. The genes implicated by MD and at least one autoimmune disease were significantly differentially expressed (both-sided test) in the substantia nigra and frontal cortex regions of the brain, and were downregulated in the liver and stomach. BD-implicated genes were not significantly differentially expressed in any specific tissue. SCZ-implicated genes were differentially expressed in the anterior cingulate cortex and the amygdala, as well as being downregulated in several other brain regions. For all tissue expression results, see Supplementary Fig. 6. Gene-set enrichment analysis of MD-implicated genes highlighted significant involvement in the Gene Ontology (GO) biological processes “response to tumor necrosis factor (TNF)” and “central nervous system development” (Table 2). For BD- or SCZ-associated genes, there was no significant enrichment in GO-related processes.

Conjunctional FDR (conjFDR) Manhattan plots illustrate genetic loci which major depression (MD) (**a**), bipolar disorder (BD) (**b**), and schizophrenia (SCZ) (**c**) share with the autoimmune diseases autoimmune thyroiditis (AITD), celiac disease (CeD), inflammatory bowel disease (IBD), multiple sclerosis (MS), psoriasis (PS), rheumatoid arthritis (RA), and type 1 diabetes (T1D). The x-axis represents chromosomal position, and the y-axis represents −log_10_ transformed FDR values. Statistically significant loci at conjFDR < 0.05 are encircled in black.

#### 3.2.3. Shared genome-wide significant genes

**Fig. 5.**
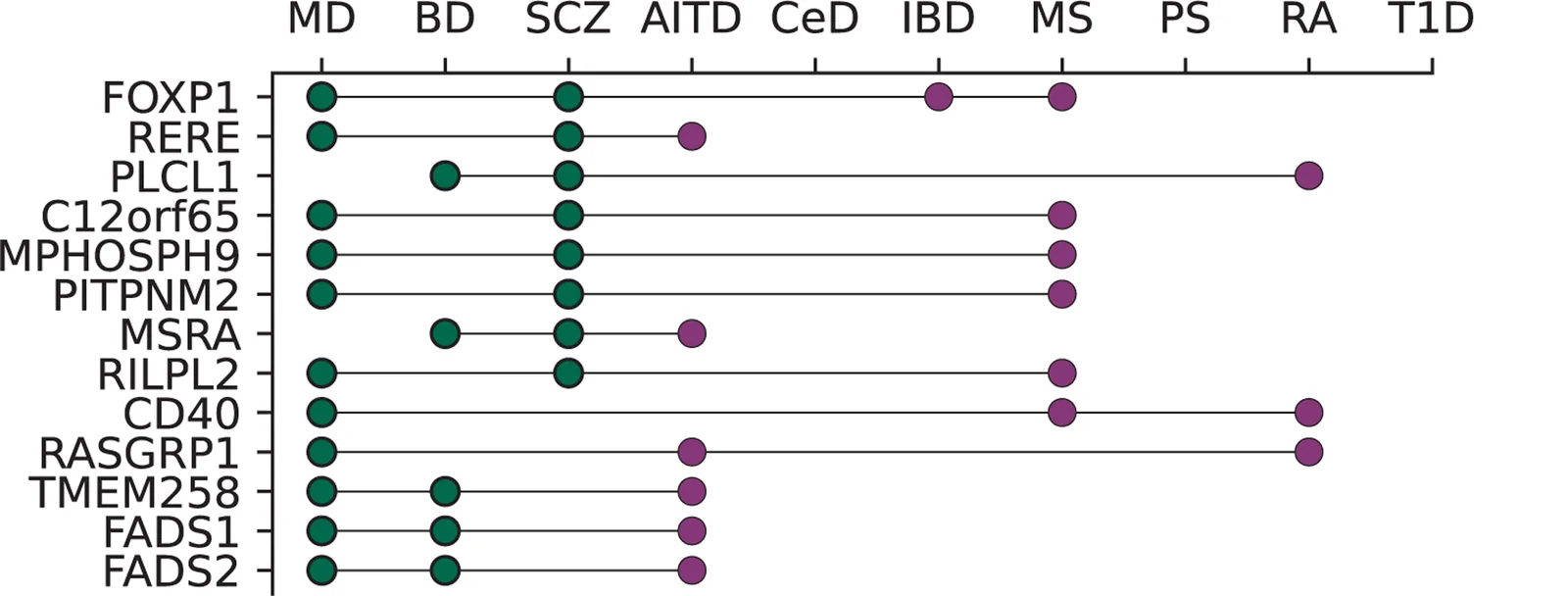
Shared genes according to MAGMA.

In addition to the genes implicated from the shared loci detected using conjFDR, we also utilized MAGMA as a complementary method to identify 78 genome-wide significant protein-coding genes jointly associated with psychiatric and autoimmune conditions (Supplementary Table 30). For an overview of the genes implicated by at least three different phenotypes, see Fig. 5 and Supplementary Table 31. The Forkhead Box P1 (*FOXP1*) gene was the only gene implicated by four disorders: MD, SCZ, IBD, and MS.

Genes identified by MAGMA significantly associated with the risk of three or more phenotypes after Bonferroni correction, including at least one of the psychiatric disorders (green dots) major depression (MD), bipolar disorder (BD), and schizophrenia (SCZ), and at least one of the autoimmune diseases (purple dots) autoimmune thyroiditis (AITD), celiac disease (CeD), inflammatory bowel disease (IBD), multiple sclerosis (MS), psoriasis (PS), rheumatoid arthritis (RA), and type 1 diabetes (T1D). For all genome-wide significant genes shared between psychiatric and autoimmune disorders identified by MAGMA, see Supplementary Table 30.

## 4. Discussion

In this genetic study, we employed a wide array of statistical methods to investigate the shared common variant architecture of the severe mental disorders MD, BD, and SCZ, and seven common autoimmune diseases. To our knowledge, this study represents the largest genomic investigation of this topic to date, involving a total of 667,518 cases from 10 GWAS datasets. By applying complementary statistical tools that measure different aspects of genetic pleiotropy, we provide a more comprehensive assessment of the shared common genetic variants across these disorders than previous work. To evaluate genetic overlap at the genome-wide level, we applied LDSC and MiXeR. Using LDSC, we found weak but significant positive global genetic correlations between MD and five autoimmune diseases: AITD, CeD, IBD, MS, and PS. In contrast, SCZ and BD were positively correlated with IBD only, indicating differences in the genetic signal shared with autoimmunity across these mental disorders. Regardless of the global genetic correlations, MiXeR revealed that a large fraction of the trait-influencing variants affecting autoimmune diseases also overlapped with mental disorders, demonstrating a higher degree of shared genetic signal than previously recognized. Finally, we provide new insights into the overlap in individual loci and genes between mental and autoimmune disorders, implicating both neurobiological and immunological mechanisms for the genes implicated for MD (Table 2). Overall, the study provides new insights into the genetic link between severe mental disorders and autoimmunity, suggesting potentially shared molecular genetic mechanisms that might inform new approaches to diagnosis and treatment of mental illness.

The LDSC analyses demonstrated weaker correlations between mental disorders and autoimmune diseases compared to the large correlations observed within the two diagnostic groups (Supplementary Fig. 1), replicating well-established results in the literature (Fominykh et al., xxxx; Lee et al., 2019). The finding that MD is more strongly correlated with autoimmune diseases diverges from previous LDSC studies based on smaller GWAS datasets, which have suggested comparable genetic correlations between the psychiatric and autoimmune conditions (Tylee et al., 2018). Our sensitivity analyses indicated that comorbid MD among patients with autoimmune diseases does not explain this relationship, as the genetic correlations were only slightly attenuated after excluding individuals with a history of depression from the autoimmune datasets.

Among the autoimmune diseases, IBD distinguished itself as the only condition genetically correlated with all three mental disorders, with positive correlations from 0.10 to 0.13, in line with recent research from our group (Smeland et al., 2025). A proposed link for a bidirectional relationship between severe mental disorders and IBD is the brain-gut axis, potentially mediated by the hypothalamus–pituitary–adrenal axis (Gracie et al., 2019; Liwayiding et al., 2025). In the most recent GWAS on BD, single-cell enrichment analysis across non-brain mouse tissues identified significant enrichment in the enteroendocrine cells of the large intestine and delta cells of the pancreas (O’Connell et al., 2025). Another noteworthy LDSC finding was the significant negative genetic correlations between BD and both RA and T1D, despite previous studies showing an increased prevalence of BD in both autoimmune diseases (Charoenngam et al., 2019; Chen et al., 2022). The fact that the common variant susceptibility for BD is positively correlated with IBD but negatively with RA and T1D further emphasizes the complex genetic relationship between BD and autoimmune diseases. This indicates that genetic variants that contribute to various autoimmune processes might either increase or decrease the genetic risk for BD. Note, however, that genetic correlations of small magnitude are unlikely to have a major impact on the comorbidity or familial aggregation of disorders. Further research is needed to clarify the clinical relevance of these associations.

The high estimated polygenicity of MD, BD, and SCZ using MiXeR is consistent with those reported for other complex brain and behavioral traits, such as cognitive ability and neuroticism (Hindley et al., 2022; Karadag et al., 2024). Conversely, the lower polygenicity estimates for autoimmune diseases are comparable to the lower end of estimates demonstrated for complex human traits (Smeland et al., 2025; Watanabe et al., 2019), akin to immunological biomarkers such as white blood cell counts and C-reactive protein level (Steen et al., 2023; Hindley et al., 2023). Regardless of whether there were significant global genetic correlations between pairs of mental and autoimmune conditions, bivariate MiXeR estimated that more than half of the trait-influencing variants linked to autoimmune diseases were also linked to psychiatric disorders in most pairwise comparisons. However, the extent of the overlap varied substantially across disorder pairs, with the proportion of overlapping autoimmune variants with psychiatric disorders ranging between 34.6–73.6 % for MD, 50.6–88.4 % for BD and 17.1–87.3 % for SCZ. Notably, CeD and IBD showed relatively larger overlap with all psychiatric disorders (range 73.2–87.3 % and 69.6–74.1 %, respectively) compared to the other autoimmune diseases. This indicates that common variants affecting immune processes related to the gastrointestinal system may influence risk of severe mental illness to a large extent, in accordance with the observed comorbidity between these disorders (Jeppesen and Benros, 2019; Marrie et al., 2019; Alkhayyat et al., 2021). Overall, these findings align with recent research from our own group, indicating that a large number of common variants have pleiotropic small effects across multiple diseases and disorders affecting different organ systems, but with a mixture of effect directions (Smeland et al., 2025; Hindley et al., 2022; Smeland et al., 2020). This suggests that shared molecular genetic mechanisms may potentially contribute to overlapping biological aspects that transcend diagnostic groups. Identifying these shared SNPs could thus reveal novel targets for improved diagnostics and personalized treatment strategies (Lee et al., 2021). For instance, gene expression analyses have shown promise in predicting non-response to antidepressants for MD patients (Cattaneo et al., 2013). Moreover, clinical studies suggest that a subgroup of individuals with depression and immune dysfunction may benefit from medications targeting the immune system (Bialek et al., 2019; Arteaga-Henríquez et al., 2019).

Using conjFDR, we identified the 172 most strongly associated shared loci between mental disorders and autoimmune diseases (Fig. 4). Approximately half of these loci were not identified in the original mental disorder GWASs, underscoring the improved statistical power and increased yield from GWASs gained by the conjFDR approach (Smeland et al., 2020). Interestingly, more than one-third of the genetic loci identified were shared by more than two conditions, suggesting pleiotropic effects across the mental and autoimmune conditions. Likewise, 13 genes identified using MAGMA and 48 correlated regions identified using LAVA were associated with at least three phenotypes. Although the discovered loci and implicated genes only account for a small portion of the shared genetic architecture between mental and autoimmune disorders, they may nevertheless provide clues into their potentially shared biology. One notable locus on chromosome 11q12.2 (top lead SNPs rs174544 and rs174564) was identified in eight separate conjFDR analyses. This locus, associated with MD, BD, and five autoimmune diseases, implicates the fatty acid desaturase (*FADS*) genes, *FADS1* and *FADS2*, both previously linked to BD (O’Connell et al., 2025). This locus was also implicated by two separate LAVA analyses, and the *FADS1* and *FADS2* genes were also significantly associated with MD, BD, and AITD according to MAGMA. The FADS enzymes play crucial roles in fatty acid biosynthesis (Merino et al., 2010). Recent investigations of the human plasma metabolome have implicated abnormal fatty acid concentrations in both mental illness and inflammatory processes (Julkunen et al., 2023), and phospholipid-related alterations in cell membranes have been hypothesized in mental disorders (Yao and van Kammen, 2004).

Another locus also linked to lipid metabolism, located on chromosome 2q33.1 (top lead SNP rs976180), was significant in six conjFDR analyses. Previously known to be associated with MD and BD (O’Connell et al., 2025; Adams et al., 2025), this locus implicates the Phospholipase C Like 1 (*PLCL1*) gene. Underscoring its importance, *PLCL1* was also significant in three separate conditions in MAGMA (BD, SCZ, and RA), and has previously been linked to both RA and the development of SCZ (Luo et al., 2022; Notaras et al., 2022). The PLCL1 protein works as a binding partner for protein phosphatase 1 and 2A, which play important roles in adipose tissue lipolysis, as well as other cellular functions, including glycogen metabolism and regulation of interferon production (Wies et al., 2013; Okumura et al., 2014; Ceulemans and Bollen, 2004). Another locus linked to protein phosphatase 1 was located on chromosome 11q13.1 (top lead SNP rs1783521), implicated in all seven conjFDR analyses for MD. This locus is associated with the Protein Phosphatase 1 Regulatory Inhibitor Subunit 14B (*PPP1R14B*) gene, which encodes an inhibitor of the serine/threonine phosphatase (Eto et al., 1999). Supporting its relevance, the locus also overlapped with a significant LAVA region.

Given the polygenic nature of mental and autoimmune disorders, it is necessary to biologically interrogate the collective set of implicated genetic variants to infer their mechanistic consequences rather than focus on individual SNPs alone, using tools such as gene-set enrichment analyses (Uffelmann and Posthuma, 2021). The lack of significant gene set enrichment for BD and SCZ likely reflects insufficient statistical power. Accordingly, larger GWAS samples are required to provide insights into the biological pathways potentially shared between BD, SCZ and autoimmune diseases. In contrast, the gene set enrichment analysis for genes implicated for MD revealed significant associations with the development of the central nervous system, a known association for both severe mental disorders and immune factors (Bennett and Molofsky, 2019; Lee et al., 2021), and also the biological pathway “response to TNF”. Note that the latter statistical association was significant despite the exclusion of the MHC region from this analysis, where the *TNF* gene itself is located. TNF, along with members of its superfamily, has important functions in the regulation of both innate and adaptive immune responses, and dysregulation of TNF signaling is linked to several autoimmune diseases (Croft et al., 2024). This finding is particularly notable given that the *TNF* gene is a drug target for infliximab, which has shown therapeutic potential in subgroups of patients with MD (Arteaga-Henríquez et al., 2019; Drevets et al., 2022).

Among the genes implicated in the TNF response pathway, two are targets for existing drugs according to the Open Targets Platform, though they have not been tested for treatment of MD; CD40 molecule (*CD40)* and TRAF3 Interacting Protein 2 (*TRAF3IP2*). *CD40* encodes a receptor expressed on B-cells which plays key roles in the adaptive immunity, for instance by interacting with CD40 ligand on activated T-cells, thereby promoting B-cell differentiation and proliferation (Elgueta et al., 2009). *TRAF3IP2* is known to be associated with PS (Ellinghaus et al., 2010), and encodes a protein which has regulatory effects on the homeostasis of B-cells by attenuating CD40 signaling (Qian et al., 2007). Underscoring its relevance, MAGMA identified *CD40* as genome-wide significant in three conditions. Also linked to TNF signaling was *FOXP1*, which was genome-wide significant in four conditions (MD, SCZ, IBD, and MS) according to MAGMA. Additionally, *FOXP1* was implicated by a genomic locus on chromosome 3p13 identified in three of our conjFDR analyses and one LAVA analysis. *FOXP1* encodes a transcription factor linked to stem cell differentiation in the bone marrow and early B cell development (Hu et al., 2006; Li et al., xxxx).

## 5. Limitations

Our study has some limitations that warrant consideration. Due to the intricate LD structure of the MHC, we were unable to precisely map the shared genetic variants in this area using the available statistical methods. However, we interpret the detected shared signal in this region as reflecting the broad involvement of the extended MHC region in both mental and autoimmune disorders, in line with previous work (Bennett and Molofsky, 2019; Pisetsky, 2023), rather than implicating any specific gene or variant, given the extensive LD and large number of genes within this locus (Horton et al., 2004). Nevertheless, our LAVA analyses showed that, across all pairwise comparisons between mental disorders and autoimmune diseases, most shared genetic regions with local correlations resided outside of this locus. LD structures outside of the MHC may also have confounded our conjFDR results, as the lead SNPs assumed to be causal might merely be in close LD with the true causal variants. Similarly, SNP associations identified by conjFDR may not represent shared genetic signals, but distinct variants in close proximity to each other. Another limitation is our investigation’s restriction to participants of European ancestry to ensure LD compatibility with genetic references. This ancestry restriction possibly limits the generalizability of our findings to other populations. Potential bias in research participation might also be a concern, for instance SCZ encompasses a wide spectrum of severity and functional outcome that could skew study representation (Lang et al., 2013). Differences in diagnostic criteria between substudies, as well as in data collection, analysis pipelines, and genotyping platforms may also potentially bias the results. For example, inclusion criteria for MD varied between physician-based diagnosis, electronic health records or self-reports (Adams et al., 2025), which may potentially show different overlap with autoimmune diseases. Related to this, differences in the unique and shared genetic architecture were observed for BD depending on the source of ascertainment as well as across clinical subgroups (O’Connell et al., 2025). New studies are needed to clarify the extent of differential overlap between mental disorders and autoimmune diseases depending on subgroups and ascertainment criteria. Lastly, while we took measures to eliminate known sample overlap, we cannot exclude the possibility that some individuals participated in multiple studies, which could potentially confound the conjFDR results.

## 6. Conclusions

This comprehensive genetic investigation shows that while severe mental disorders and autoimmune diseases have distinctly different genetic architectures, they also exhibit notable overlap, involving hundreds of common genetic variants with a mixture of effect directions. Our study highlights several genes that warrant further examination, particularly genes involved in lipid metabolism and TNF signaling.

## Supplementary Material

1

2

## Figures and Tables

**Table 1:** Overview GWASs used in the study.

Phenotype	Abbr.	N cases	N controls	Ancestry	Consortium	GWAS source, PMID

Major depression	MD	412,305	1,588,397	European	PGC	37464041 (Adams et al., 2025)
Bipolar disorder	BD	59,287	781,022	European	PGC	39843750 (O’Connell et al., 2025)
Schizophrenia	SCZ	53,386	77,258	European	PGC	35396580 (Trubetskoy et al., 2022)
Autoimmune thyroiditis	AITD	30,234	725,172	European	deCODE	32581359 (Saevarsdottir et al., 2020)
Celiac disease	CeD	10,573	1,077,290	European	META	UK Biobank, FinnGen (Kurki et al., 2023); MoBa (Magnus et al., 2016), HUSK (Refsum et al., 2006)
Inflammatory bowel disease	IBD	25,042	34,915	European	IIBDGC	28067908 (de Lange et al., 2017)
Multiple sclerosis	MS	14,802	26,703	European	IMSGC	31604244 (International Multiple Sclerosis Genetics Consortium, 2019)
Psoriasis	PS	20,597	1,077,700	European	META	UK Biobank, FinnGen (Kurki et al., 2023), MoBa (Magnus et al., 2016), HUSK (Refsum et al., 2006)
Rheumatoid arthritis	RA	22,350	74,823	European	META	36333501 (Ishigaki et al., 2022)
Type 1 diabetes	T1D	18,942	501,638	European	META	34012112 (Chiou et al., 2021)

Abbreviations: PubMed Identifier (PMID), Psychiatric Genomics Consortium (PGC), Norwegian Mother, Father and Child Cohort Study (MoBa), Hordaland Health Studies (HUSK), *meta*-analysis (META).

**Table 2:** Major depression associated genes involved in GO biological processes.

Biological process	p-value	Adjusted p-value	Genes involved

Response to tumor necrosis factor	6.87e-6	3.30e-2	*TANK, FOXP1, TRAF3IP2, LRRK2, CD40, SPATA2*
Central nervous system development	8.51e-6	3.30e-2	*FOXP1, LRRK2, RERE, TAL1, ELAVL4, SLC8A1, NR4A2, OLIG3, IMMP2L, KDM2B, BCL11B, SEMA6D, BPTF*

Significant Gene Ontology (GO) biological processes associated with genes jointly associated with major depression and at least one of the autoimmune diseases autoimmune thyroiditis, celiac disease, inflammatory bowel disease, multiple sclerosis, psoriasis, rheumatoid arthritis, and type 1 diabetes. Genes analyzed are identified by Open Targets Genetics (https://genetics.opentargets.org/) as the top genes associated with shared genetic loci between MD and the autoimmune phenotypes identified at conjFDR < 0.05. See Supplementary Table 29 for gene summary of the implicated genes.

## Data Availability

GWAS summary statistics are publicly available at GWAS catalogue or available on request (International Multiple Sclerosis Genetic Consortium, HUSK, MoBa)

## Appendix A. Supplementary data

Supplementary data to this article can be found online at https://doi.org/10.1016/j.bbi.2025.106249.
